# Cell Fusion and Syncytium Formation in Betaherpesvirus Infection

**DOI:** 10.3390/v13101973

**Published:** 2021-09-30

**Authors:** Jiajia Tang, Giada Frascaroli, Xuan Zhou, Jan Knickmann, Wolfram Brune

**Affiliations:** 1 Leibniz Institute for Experimental Virology (HPI), 20251 Hamburg, Germany; 184283@shsmu.edu.cn (J.T.); giada.frascaroli@leibniz-hpi.de (G.F.); xuan.zhou@leibniz-hpi.de (X.Z.); jan.knickmann@leibniz-hpi.de (J.K.); 2 Center for Single-Cell Omics, School of Medicine, Shanghai Jiao Tong University, Shanghai 200025, China

**Keywords:** cell–cell fusion, syncytium formation, polykaryocyte, *Herpesviridae*, herpesvirus, cytomegalovirus, envelope glycoproteins, glycoprotein B, glycoprotein H, glycoprotein L

## Abstract

Cell–cell fusion is a fundamental and complex process that occurs during reproduction, organ and tissue growth, cancer metastasis, immune response, and infection. All enveloped viruses express one or more proteins that drive the fusion of the viral envelope with cellular membranes. The same proteins can mediate the fusion of the plasma membranes of adjacent cells, leading to the formation of multinucleated syncytia. While cell–cell fusion triggered by alpha- and gammaherpesviruses is well-studied, much less is known about the fusogenic potential of betaherpesviruses such as human cytomegalovirus (HCMV) and human herpesviruses 6 and 7 (HHV-6 and HHV-7). These are slow-growing viruses that are highly prevalent in the human population and associated with several diseases, particularly in individuals with an immature or impaired immune system such as fetuses and transplant recipients. While HHV-6 and HHV-7 are strictly lymphotropic, HCMV infects a very broad range of cell types including epithelial, endothelial, mesenchymal, and myeloid cells. Syncytia have been observed occasionally for all three betaherpesviruses, both during in vitro and in vivo infection. Since cell–cell fusion may allow efficient spread to neighboring cells without exposure to neutralizing antibodies and other host immune factors, viral-induced syncytia may be important for viral dissemination, long-term persistence, and pathogenicity. In this review, we provide an overview of the viral and cellular factors and mechanisms identified so far in the process of cell–cell fusion induced by betaherpesviruses and discuss the possible consequences for cellular dysfunction and pathogenesis.

## 1. Introduction

Cell–cell fusion is a process in which the plasma membranes of two adjacent cells merge into a single continuous membrane bilayer. This results in a mixing of the luminal contents and the formation of bi- or multinucleated cells termed syncytia [1]. Generally, cell–cell fusion occurs rather infrequently under physiological conditions. However, it plays a fundamental role in the development and physiology of multicellular organisms: the fusion of spermatocyte and oocyte is required for fertilization, and the fusion of trophoblast cells results in the formation of the syncytiotrophoblast, a layer that extends over the surfaces of all villous trees and represents the outermost layer of the human placenta. The fusion of myoblasts gives rise to muscle fibers, and the fusion of bone macrophages results in multinucleated osteoclasts that are indispensable for bone homeostasis [2]. In contrast to these physiological functions, an increasing number of studies have shown that cell–cell fusion can drive cell transformation and cancer progression by impairing genetic stability, promoting metastasis, and contributing to drug resistance [3,4]. Moreover, multinucleated cells are found in various granulomatous diseases (including tuberculosis, leprosy, schistosomiasis, and sarcoidosis) as a macrophage response to the chronic inflammatory milieu induced by different bacteria and protozoa [5,6,7,8].

**Figure 1:**
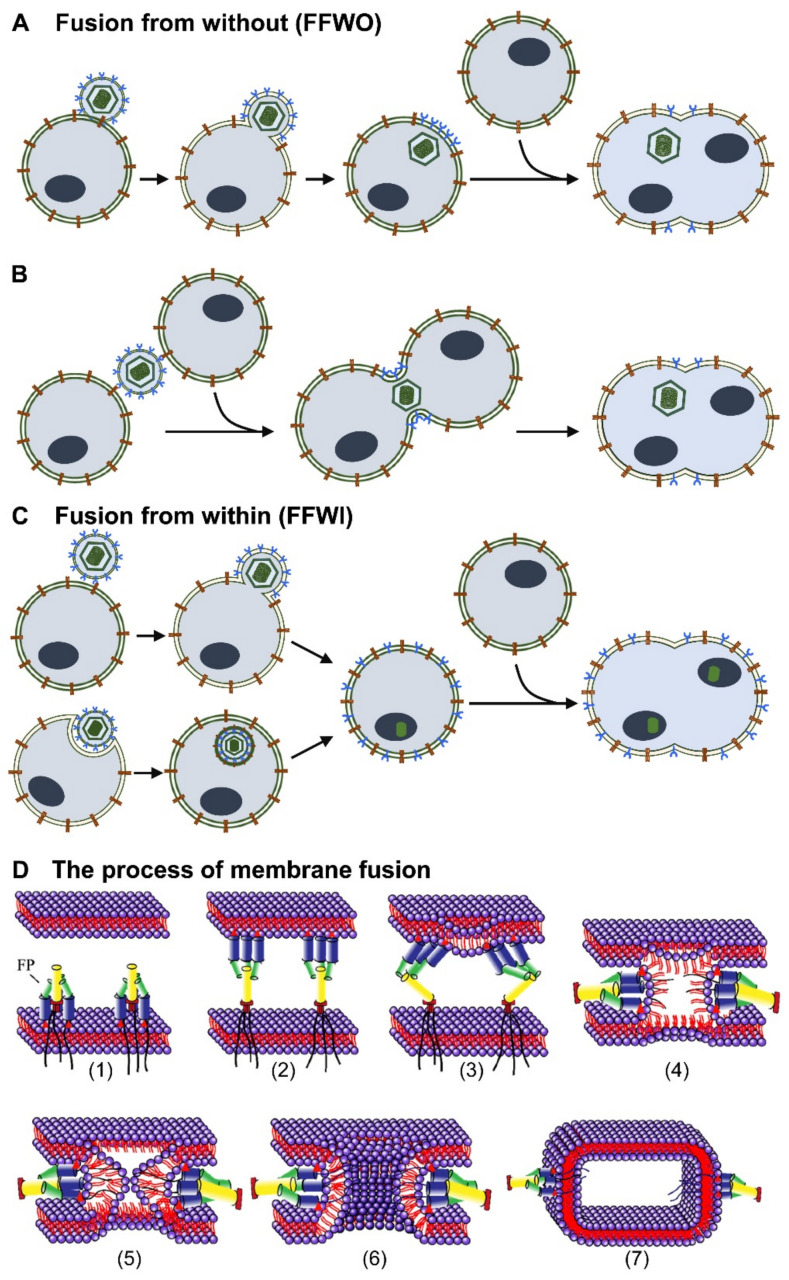
Simplified view of the different types of virus-induced membrane fusion. (**A**) Fusion from without (FFWO): The virus envelope carries glycoproteins, mediating the fusion of the viral envelope with the plasma membrane. The viral envelope is retained on the surface of the infected cell. Envelope glycoproteins interact with receptors on neighboring cells and mediate cell–cell fusion. (**B**) FFWO can also occur when a viral particle fuses simultaneously with two cells. (**C**) Fusion from within (FFWI): The virus enters the cell through fusion with the plasma membrane or through endocytosis. Viral gene expression leads to the synthesis of envelope glycoproteins that may be transported to the cellular surface. Viral glycoproteins interact with receptors on adjacent cells and induce cell–cell fusion. (**D**) Schematic of the membrane fusion process. (1) Activation of the fusion machinery and exposure of specific fusion peptides (FP). (2) Insertion of the FP into the adjacent membrane. (3) Refolding of the FP and induction of membrane deformation. (4) Formation of a transient hemifusion diaphragm. (5) Opening of a fusion pore that completes merging of both membranes. (6) Expansion of the fusion pore. (7) Situation after membrane fusion (postfusion).

All enveloped viruses necessarily express proteins that mediate the fusion of the viral envelope with membranes of the target cell, either at the plasma membrane or inside endocytic vesicles. Besides their presence in the viral envelope, fusogenic proteins are synthesized during the viral replication cycle and traffic to the cellular membranes prior to being incorporated into budding new viral particles. When the number of fusogenic proteins decorating the infected cell is sufficient to engage receptors on neighboring cells, cell–cell fusion may be triggered, resulting in syncytium formation (Figure 1) [9,10,11,12]. Many enveloped viruses belonging to different families of human pathogens mediate cell–cell fusion to enhance viral spread and persistence even in the presence of antiviral effector responses or in the absence of extracellular virions. For example, respiratory viruses such as human respiratory syncytial virus, measles virus, influenza viruses, and SARS-coronaviruses that enter the human body through the epithelial lining of the airways use cell–cell fusion as a strategy to overcome the mucociliary blanket, which contains antibodies and mechanically traps viral particles [13,14]. Thus, cell–cell fusion allows these viruses to spread more efficiently in the lung. Moreover, the process of syncytium formation constitutes a hallmark of human immunodeficiency virus (HIV) infection in humans as well as in monkey and mouse models [15]. HIV-infected syncytia, mainly formed by the fusion of monocytes, lymphocytes, and dendritic cells, have been detected in the brain and lymphoid organs of HIV-infected individuals [16]. In all cases, syncytium formation has been linked to viral pathogenesis, destruction of cellular function, and increased disease severity [17,18].

By allowing the delivery of virus genomes directly to the new target cell rather than being randomly released into the extracellular milieu, cell–cell fusion promotes fast viral spread within a tissue, prevents viral exposure to humoral immunity, and probably also allows the infection of cells not expressing the specific entry receptor/s. For example, it has been reported for Epstein–Barr virus (EBV) that viral genomes can be transferred by fusion to epithelial cells devoid of EBV receptors [19]. Even though the specific composition, mechanism of formation, and physiologic role of these virus-induced syncytia may vary depending on the virus, the cellular targets, and the tissue microenvironment, virus-induced syncytia may share some common properties. When analyzed in vitro, these infected multinucleated cells exhibit extended survival, improved motility, and high capacity of viral production, suggesting that they are important virulence and pathogenicity factors [20,21]. On the other hand, although some viruses have acquired the ability to exploit cell–cell fusion for their own benefit, the formation of syncytia remains a tightly regulated process that can also be detrimental for a virus. For example, the fusion of dividing cells leads to the formation of polyploid cells that can undergo premature cell death due to cytoskeletal aberrations or mitotic catastrophe [22], and these consequences can be detrimental or even lethal for viruses that replicate slowly or persist for a prolonged period of time. In conclusion, syncytium formation appears to represent a high degree of viral adaptation to its host, and therefore a more thorough understanding of this phenomenon can help in dissecting the viral fusion machinery and explain viral pathogenesis. Finally, it can also be of practical use for new therapeutic strategies targeting viral cell-to-cell spread, for reducing the dysfunction of fused cells as suggested for COVID-19 [23], or eventually for the design of optimized oncolytic viruses [24].

Unlike other enveloped viruses such as HIV, hepatitis C virus (HCV), and Ebola virus (EBOV) that usually employ a single envelope protein to mediate the fusion of the viral envelope with host cell membranes, herpesviruses are equipped with a complex set of proteins that execute the fusion of the viral envelope with the cellular membrane. A core fusion machinery, formed by the glycoproteins B, H, and L (gB, gH, and gL, respectively) and a number of additional accessory proteins that provide cellular tropism and/or modulate the conformation and fusogenic activity of the core fusion machinery [25]. These fusogenic and accessory regulatory glycoproteins function in a tightly controlled and ordered manner, making the process of virus–cell fusion very flexible and adaptable to the specific cellular target and physiologic conditions [13]. In this review, we describe the general principle of membrane fusion, the herpesviral glycoproteins involved in cell fusion, and mutants/variants associated with increased fusogenicity, and we also discuss the potential role of hyperfusogenic variants for pathogenesis and disease in humans focusing on the betaherpesviruses.

## 2. Syncytium Formation by Herpesviruses

The Herpesviruses (*Herpesviridae*) are a family of large enveloped DNA viruses that infect reptiles, birds, and mammals. To date, nine human herpesviruses (HHV) have been identified and classified into three subfamilies, the *Alpha*-, *Beta*-, and *Gammaherpesvirinae* [26]. The human α-herpesviruses, which include herpes simplex virus type 1 and type 2 (HSV-1 and HSV-2) and varicella zoster virus (VZV), are fast-growing cytolytic viruses that infect a wide variety of cell types but preferentially infect the neurons of the peripheral nervous system where they establish latent infections [27]. HSV-1 and HSV-2 are the prototypical herpesviruses usually associated with localized mucocutaneous lesions in the oral or genital regions. VZV primary infection usually causes varicella (chicken pox) while VZV reactivation causes zoster (shingles). The human β-herpesviruses include human cytomegalovirus (HCMV, HHV-5) and the roseoloviruses HHV-6A, 6B, and 7. They are slowly growing viruses and exhibit different cellular tropism. While HHV-6 and HHV-7 are mainly lymphotropic, HCMV infects many different cell types: epithelial and endothelial cells, fibroblasts, and myeloid cells, but not lymphocytes. HCMV is the prototype of the betaherpesviruses, has the largest double-stranded DNA genome of all human viruses, and encodes up to 200 genes and an even larger number of polypeptides. HCMV generally causes inapparent or mild infections in otherwise healthy individuals. However, in immunocompromised patients, such as transplant recipients or AIDS patients, uncontrolled HCMV infection can lead to various diseases, such as pneumonitis, hepatitis, colitis, esophagitis, and retinitis. It has also been linked to certain forms of vascular disease and cancer [28]. Another major clinical problem caused by HCMV is congenital infection, and despite low public awareness, HCMV is the leading infectious cause of birth defects [29]. Primary infection of the mother during pregnancy results in a 30% to 40% chance of transmission to the fetus, and approximately 15% of infected newborns will suffer from acute disease or late sequelae, such as sensorineural hearing loss, blindness, epilepsy, mental retardation, or microcephaly [29].

While very little is known about the clinical relevance of HHV-6A, HHV-6B and (less frequently) HHV-7 are the causative agent of exanthema subitum (also known as roseola infantum or sixth disease) and have been associated with certain epilepsy syndromes such as febrile seizures and certain forms of encephalopathy in immunosuppressed patients [30].

The human gammaherpesviruses include Epstein–Barr virus (EBV, HHV-4) and Kaposi sarcoma-associated herpesvirus (KSHV, HHV-8), which cause severe diseases in immunocompromised individuals such as transplant recipients and HIV-infected patients and are the only human herpesviruses with a well-established role in carcinogenesis. EBV is associated with nasopharyngeal carcinoma, Burkitt’s lymphoma, and certain forms of Hodgkin’s lymphoma [31], while KSHV causes Kaposi’s sarcoma (an endothelial cell neoplasm), primary effusion lymphoma, and multicentric Castleman’s disease [32].

Although syncytium formation by herpesviruses has not gained much attention so far, all herpesviruses have been shown to be capable of forming syncytia during natural or in vitro infection [19,33,34,35,36,37,38,39,40]. Syncytium formation has been commonly reported for VSV, the most fusogenic herpesvirus, as well as for HSV and EBV, and several review articles covering these viruses have been published elsewhere [39,41,42,43]. During VZV infection, the presence of extensive syncytia in skin lesions as well as in the sensory ganglia is not only a hallmark of infection but also a diagnostic parameter [44,45]. Importantly, syncytium formation between VZV-infected satellite cells and neurons in the ganglia is thought to cause functional aberrations implicated in postherpetic neuralgia, a painful condition that remains difficult to treat [46]. Microscopic visualization of tissues from the lesions of HSV-infected patients typically reveals syncytia [47,48]; however, many patient isolates do not induce syncytia or only induce syncytia to a limited extent in tissue culture [39]. For HSV, the size and abundance of syncytia are highly variable depending on the strain-specific variants of viral envelope glycoproteins involved in cell entry [42,49]. Hypersyncytial HSV mutants, capable of forming more extensive syncytia and in cell types that usually are not fused by HSV can also arise in culture and frequently contain mutations in the viral genes encoding glycoproteins gB and gK [42]. Syncytium formation has been observed in Epstein–Barr virus-superinfected Raji cells [19], and it has been suggested that fusion of EBV-carrying cells with epithelial cells may be the mode of entry of the virus into cells unable to absorb the virus otherwise [34].

Although syncytium formation by betaherpesviruses such as HCMV, HHV-6, and HHV-7 has been observed [33,50], it has not received much attention, and even though HCMV-induced syncytia may have clinical relevance in vivo, they remain largely uncharacterized. Syncytia associated with HCMV infection have been observed during isolation and cultivation of congenital strains in epithelial but not endothelial cells [51,52,53,54] and upon emergence of specific mutations in viral envelope glycoproteins [55]. Among the cytomegaloviruses infecting animals, the closest homologs of HCMV are the chimpanzee and rhesus CMVs (RhCMV). The latter has been studied more extensively and is used as a non-human primate model for studies of pathogenesis and vaccine development [56]. Syncytium formation has been reported for RhCMV in cell culture and in tissues of RhCMV-infected animals [57]. Syncytium formation has also been reported for mouse as well as rat CMV [58,59,60] and has been observed in the brains of suckling rodents upon intracerebral infection [61,62]. Formation of large polykaryocytes has been reported for susceptible human T lymphocytes upon HHV-6 infection [63] as well as in phytohemagglutinin-stimulated peripheral blood mononuclear cells (PBMC) upon infection with HHV-7 [64]. For betaherpesviruses, syncytium formation depends not only on strain-specific variants of viral envelope proteins but also on the specific cell type undergoing viral infection [39,65,66], thus demonstrating that virus-induced cell–cell fusion is a very complex mechanism involving both viral and cellular factors.

## 3. Determinants and Mechanism of Syncytium Formation

Cell–cell fusion requires the merging of two adjacent plasma membranes, an energy unfavorable process, that proceeds through a stalk–hemifusion–pore model and that requires the action of specific fusion proteins that lower the energy barrier of the process, maintain contact, and finally merge the proximal and distal leaflets of the two membranes. When driven by viral fusion machineries, the process of membrane fusion involves several sequential steps (Figure 1D): (1) the activation of the fusion machinery, which exposes a fusion peptide (FP); (2) the insertion of the FP into the adjacent membrane, and approaching the two opposed membranes from a very close distance (e.g., 10–20 nm); (3) the refolding of the fusion protein, which induces membrane deformation; (4–5) the formation of the stalk, where only the outer leaflets of the membranes merge and locally the membrane lipidic content mix; (6) the expansion of the stalk and formation of a transient hemifusion diaphragm with intact distal leaflets; and (7) the opening of a fusion pore that completes merging of both membranes and mixing also of the aqueous content [1,67,68,69,70]. Most viral fusogens are organized as homo- or hetero-oligomeric complexes comprising one (e.g., influenza virus) or up to four (e.g., herpes simplex virus) types of transmembrane envelope glycoproteins. Premature activation of the fusogen and incorrect fusion can be deleterious for the virus and are therefore avoided by maintaining the fusogen in a suppressed state until the timing and location of membrane fusion assure a productive (i.e., leading to infection) deployment of the fusion machinery. In the absence of optimal conditions, the fusogen resides in a metastable prefusion state on the viral membrane in an inactive conformation achieved either by positioning of the fusion domains buried inside the protein or by inhibitory interactions with peripheral subunits or accessory proteins [71,72,73].

Environmental signals such as virus interaction with cell-surface receptors or chemical modification upon exposure to mildly acidic pH along the endocytic pathway induce conformational changes in the fusogen, release the inhibition, and trigger the exposure of their previously shielded fusion protein in the direction of the opposing membrane. The fusogenic conformation is often elongated, unstable, and folds back on itself rapidly so that the portion of the fusogen anchored to the viral envelope and the fusion segment inserted into the opposing membrane come close together. In addition, the fusion machinery is also spatially regulated, as membrane fusion requires a critical number and positioning of fusogenic proteins. Viral fusogenic proteins can reach the cellular plasma membrane in two different ways. In the first, the glycoproteins present on the viral envelope are simply transferred to the cell membrane during the process of virus entry (Figure 1A, B). In this situation, the viral fusion proteins decorating the cell surface can interact with the cognate receptors on the plasma membrane of a neighboring cell and drive a type of fusion called fusion from without (FFWO). FFWO depends on temperature and pH and requires a high concentration of viral particles but does not require newly synthesized viral gene products [74]. FFWO can be difficult to observe experimentally because the physical and chemical properties of the fusogenic proteins present in the viral envelope are often altered by the centrifugation steps that are required to obtain the high-titer viral stocks necessary for infection experiments at high multiplicities of infection. In the second form of viral-induced cell–cell fusion, the virus enters cells through membrane fusion or endocytosis and initiates viral gene expression. Newly synthesized viral glycoproteins are transported to the cell membrane and mediate fusion with neighboring cells (Figure 1C). This type of fusion is called fusion from within (FFWI) [74]. Since it requires the accumulation of newly synthesized viral proteins, it can occur hours or days after infection. It can also be studied in vitro upon ectopic expression of fusogenic proteins in transfected cells. Independent of the type of fusion, the set of viral glycoproteins required for cell–cell fusion seems to be conserved between alpha-, beta-, and gammaherpesviruses. It is now widely accepted that cell–cell fusion by herpesviruses requires a set of conserved glycoproteins (i.e., gB, gH, and gL) that constitute the core fusion machinery and a set of accessory proteins that vary considerably between the different herpesviruses [73,75,76,77,78].

HCMV, the prototype of the betaherpesviruses, encodes seven glycoproteins critical for viral entry and membrane fusion: gB, gH, gL, gO, UL128, UL130, and UL131A [79,80,81]. Glycoprotein B (gB), gH, and gL are conserved among the herpesviruses and are essential for infectivity [75]. Co-expression of the glycoproteins that make up the fusion machinery induces cell–cell fusion and the formation of extensive syncytia [36,55,82,83]. Glycoprotein B alone can mediate fusion only in certain cases. For instance, stable expression of HCMV gB alone can induce syncytia in U373 glioblastoma cells but not in other cell types [84]. Normally, HCMV gB requires the assistance of the conserved gH–gL complex to induce fusion of two adjacent membranes [85]. gH and gL are implicated in membrane binding and induce the activation of gB fusogenic activity by imposing conformational changes on gB. Therefore, gB, gH, and gL together constitute the core fusion machinery of herpesviruses. Additional accessory proteins characteristic of alpha, beta, or gammaherpesviruses provide additional layers of regulation and assure a specific adaptation of the virus to its target cells. For HCMV, the accessory proteins involved in cell–cell fusion include the three products of the *UL128* locus (UL128, UL130, and UL131A), gO, gM and gN, and finally the chemokine receptor US28.

### 3.1. The HCMV Core Fusion Machinery: Glycoprotein gB, the HCMV Fusogen

On the basis of their pre- and post-fusion structures, viral fusogens have been divided into three classes: (i) class I fusogens, which include, for instance, hemagglutinins from influenza viruses and are dominated by α-helical coils; (ii) class II fusogens, which include glycoprotein E from Dengue virus and consist predominantly of β-sheets; and (iii) class III fusogens, which include the vesicular stomatitis virus (VSV) G protein and gB of herpesviruses and feature both types of secondary structures [71,73].

**Figure 2:**
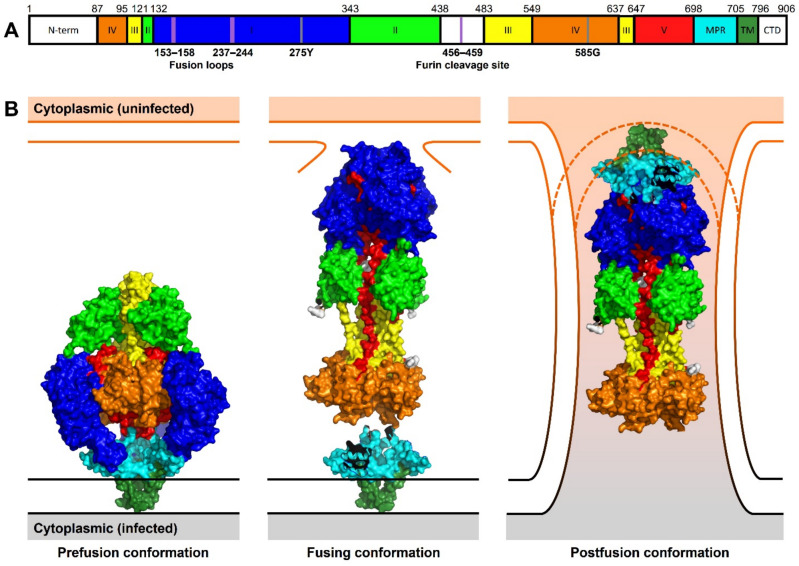
Linear representation and structural conformations of HCMV gB. (**A**) Distribution of HCMV gB structural domains within the gB primary domain. Amino acid indices at the domain boundaries are indicated above the sequence representation. Fusion loops and furin cleavage site are indicated as violet lines. Two known residues that effect HCMV gB fusion activity are marked as grey lines. (**B**) gB protein structures indicating conformations during the stages of cell–cell fusion. The prefusion conformation (PDB 7KDP) is compressed on the infected cell membrane. During cell–cell fusion, gB extends to bind to an additional uninfected cell membrane. The fusing conformation (PDB 5CXF) is modeled with the MPR-TM domain (PDB 7KDP) modeled by protein alignment to the conserved IV domain. In the postfusion conformation, gB (PDB 5CFX) is modeled with the MPR-TM domain (PDB 7KDP) as shown in [92]. 2021. The surface representation has been generated using PyMOL and the structural domains are mapped and color-coded as described [90,92]: N-terminal signaling sequence (N-term) and cytoplasmic domain (CTD) in white; domain I = blue, II = green, III = yellow, IV = orange, V = red; membrane proximal region (MPR) in cyan, and transmembrane region (TM) in dark green.

HCMV gB, encoded by ORF *UL55*, the most abundant glycoprotein present on the HCMV envelope [86], is a type III viral fusogen and as such essential for HCMV entry into target cells and for cell–cell fusion [87,88]. It is synthesized as a 906 amino acid (aa) precursor protein of approx. 160 kDa, which is glycosylated and proteolytically cleaved between residues 460 and 461 by the cellular enzyme furin. Cleavage generates two fragments of 116 and 55 kDa that remain disulfide-linked to each other [73]. gB is highly immunogenic in natural infection and has been studied extensively as a dominant target of virus-neutralizing antibodies [89]. Each full-length gB molecule (Figure 2A) contains a short N-terminal region with a signal peptide (aa 1−87), a large ectodomain with five distinct antigenic domains (domain I to V; aa 1–705), a hydrophobic membrane-proximal region (MPR; aa 706–751), a transmembrane domain (TM; aa 752−796), and finally a cytoplasmic domain (Cyto; aa 797–906) [90,91,92].

Although the fusogenic activity of gB derives from its ectodomain, the other domains (MPR, TM, and Cyto), which make up 20% of the full-length protein, are thought to play key roles in fusion regulation [93]. Especially important for the fusogenic activity of herpesviral gB seems to be the cytoplasmic domain because truncations, point mutations, or insertions in this domain have been shown to induce hyperfusogenic phenotypes in HSV, VZV, and EBV infections [94,95,96,97,98,99]. In contrast, point mutations in HCMV gB’s predicted sorting motifs (tyrosine and dileucine motifs at position 845/894 and 883/884, respectively) failed to produce a hyperfusogenic form [100]. Only HCMV gB chimeras in which the large Cyto, TM, and a portion of the MPR of gB are replaced with the unstructured and short TM and cytoplasmic domain of VSV G exhibit constitutive membrane fusion capacity and induce the formation of large multinucleated syncytia in different cell types [100,101]. Taking also into account the three-dimensional structure of gB (Figure 2B), one possible explanation for these regulatory properties is that the Cyto functions as a “clamp”, controlling the fold of gB and stabilizing it in its prefusion conformation. gB indeed exists in two alternative conformations that have been resolved to a few Å resolution by cryo-electron microscopy or X-ray crystallography: a low-energy post-fusion conformation [90,91,102,103] and a high-energy pre-fusion conformation [92] (Figure 2B). Prefusion gB forms a compact structure, a tripod of approximately 100 Å in diameter and 110-30 Å long, whereas post-fusion gB forms an elongated spike-like hairpin with a diameter of 70 Å and is roughly 170 Å tall [90,102,103]. While in its pre-fusion conformation, the fusion loops are located at the base of the trimer in a position close to the viral membrane and tucked away from the target membrane, in the post-fusion conformation, gB appears elongated in the direction of the target membrane with three central helices pushing the FP together in the direction of target membrane. It has been proposed that while the hydrophobic residues of fusion loops penetrate the membrane, the positively charged central groove can interact with negatively charged phospholipid heads, thereby mediating cell fusion in a synergistic manner (Figure 2B) [92].

According to the current model, it is the conformational change between the pre- to the post-fusion form of gB that mediates the exposure of the fusion loops and the merger of the two membranes. Fusion loops are usually rich in hydrophobic residues that are important for membrane insertion and penetration [104]. Two fusion loops have been identified in HCMV gB, 153YAYIYT158 and 237GSTWLYRE244, and mutagenesis experiments have shown that the substitution of hydrophobic residues 155YIY157 and W240 can abolish fusion, confirming their functional importance [105].

Glycosylation is an important post-translational modification that affects proteins’ folding, stability, and intracellular trafficking, thus influencing the full spectrum of biological functions. HCMV gB contains 18 N-linked glycosylation sites mainly located in DI and DII [106]. On the one hand, glycosylation provides a shield of glycans to protect functional domains from neutralizing antibodies. On the other hand, glycosylation is also required for cell fusion activity. Mutagenesis experiments with HSV-2 gB have shown that mutation of specific N-glycosylation sites reduces the fusogenicity of gB and leads to decreased fusion activities [107]. Consistently, a glycosylation inhibitor interfered with syncytium formation in U373 glioblastoma cells that stably express HCMV gB [84]. Since the glycosylation processes can be very cell-type-dependent and different sugar moieties can be added to the same protein in different cell types, studies with gB should ideally be conducted in several different cell types. Altogether, these studies suggest that mutations at the glycosylation sites can affect the intracellular trafficking and transport of gB, thus resulting in lower level of gB at the cell surface [107] or impaired protein trimerization/folding, thus affecting gB interaction with other glycoproteins or receptors [107].

An alignment of more than 60 gB sequences in clinical and laboratory HCMV isolates revealed a conservation score of 88% to 99%. The regions with the greatest diversity lie within the N-terminal signal peptide, the disordered domain II loop, and the crown of domain IV [90]. Recent work has demonstrated that unique gB variants in strains AD169 and VR1814 (gB(275Y) and gB(585G), respectively) account for increased fusogenicity, faster viral entry, and the formation of large multinucleated syncytia [108]. In these two strains, a single amino acid residue of gB determined the entry mode of HCMV. While AD169 gB(275Y) entered fibroblasts rapidly, probably by direct fusion at the plasma membrane, AD169 gB(275D) entered more slowly, probably by macropinocytosis, since this delayed entry was sensitive to a macropinocytosis inhibitor. Since gB cooperates with the trimeric gH–gL–gO and the pentameric gH–gL–UL128–UL130–UL131A complexes for entry, the increased fusogenicity of AD169 gB(275Y) might either be the result of an inherently hyperfusogenic gB or an altered interaction of gB with the other components of the fusion machinery. The highly fusogenic gB(275Y) variant is present in all sequenced AD169 variants; however, since the original clinical sample that led to the isolation of strain AD169 in 1956 is no longer available, it remains unclear whether the gB(275Y) variant was present in the original virus or arose later during cell culture adaptation. The same holds true for the gB(585G) variant of strain VR1814, which has also been passaged many times in cell culture. Interestingly, the presence of the gB(275Y) variant in HCMV isolates N12 and UCSF-1a (GenBank CAA07368 and AZB79941) and gB(585G) in isolates P4, P14, and P15 (Genbank QPZ44673, QPZ45165, and QPZ45328) suggests that highly fusogenic gB variants exist in human patients. Moreover, a recent study reported syncytium-forming phenotypes among clinical HCMV isolates from congenitally infected infants [54]. This raises the intriguing question of whether syncytium-forming HCMV strains might be associated with increased transmission or pathogenicity.

### 3.2. The HCMV Core Fusion Machinery: gH and gL, the HCMV Fusion Trigger

Glycoproteins gH and gL are conserved among herpesviruses and present in the viral envelope as a stable complex. gH is an 86 kDa protein encoded by the *UL75* gene. Two genotypes have been described based on the genetic variability in the N-terminal domain [109]. gL is a 30 kDa glycoprotein encoded by the highly conserved ORF *UL115*. The large ectodomain of gH is anchored to the membrane by a single C-terminal transmembrane anchor, and gL is associated to gH’s ectodomain. Unlike the gH–gL complexes of HSV or EBV, HCMV gH–gL is a stable heterodimer covalently linked by a disulfide bond between residues gH-C95 and gL-C47 [110]. The crystal structure of HCMV gH–gL has not yet been determined, but the structure has been predicted based on EM data and the sequence and functional conservation between herpesviruses. These calculations predicted an “L”-shaped or boot-shaped spike protruding outwards from the viral envelope [85,91,111,112].

Since the expression of HCMV gH–gL alone without gB caused syncytia in certain cell types, it was initially hypothesized that gH–gL could have inherent fusogenic properties and might function as a co-fusogen of gB [44,76,83]. However, structure analysis of gH–gL showed a novel architecture that did not resemble any known viral fusion protein [85,91,111], and fusion was achieved only in a selected set of cells. Thus, it has become accepted that gH–gL probably acts as a regulator of fusion rather than a co-fusogen with gB. It is currently broadly accepted that gH–gL regulates cell fusion through driving gB conformational change from the pre-fusion to the post-fusion conformation [73,75,113].

According to the current model, the activation of gB fusogenic activity includes a sequential series of steps: first, the engagement of a herpesviral receptor-binding protein such as gO, UL128, UL130, or UL131A with the cellular receptor, then the signal transduction to gH–gL, and finally the release of inhibitory forces on gB [110,114]. For HCMV, only ~7% of gH–gL complexes present on the viral envelope are in contact with the prefusion gB trimer, while more than 90% are unbound, thus supporting the notion that for binding with gB, gH–gL complexes need additional modifications such as the binding with viral fusion accessory protein gO or the three products of the *UL128–131* genetic locus [91].

### 3.3. The HCMV Accessory Proteins Involved in Membrane Fusion

Of the more than twenty viral proteins that have been recognized as structural components of the viral envelope [86,115], only few have been implicated in the process of virus entry and/or cell–cell fusion. By far the most important accessory proteins are glycoprotein O (gO) and the three proteins encoded by ORFs *UL128*, *UL130*, and *UL131A*. These proteins compete for the association with gH–gL. On the virion envelope, they can be found either as a trimeric gH–gL–gO complex or as a pentameric complex formed by gH–gL–UL128–UL130–UL131A [116,117]. Unlike gL and gH, none of these accessory proteins are absolutely necessary for viral infection and growth because viruses lacking gO or UL128–UL131A can still replicate, spread, and release viral particles into the extracellular milieu. gO is a non-essential but replication-enhancing protein and plays a critical role in the secondary envelopment and release of cell-free virions [118,119,120]. gO is a highly glycosylated 125-kDa glycoprotein encoded by the ORF *UL74* of HCMV and shares 40% similarity and 20% identity at the amino acid level with the positional homologs found in all other betaherpesviruses [121]. The *UL74* ORF is one of the most variable loci in the HCMV genome, and genetic analyses in patients’ isolates have led to the definition of eight different genotypes [118]. So far, there is no evidence for an association between syncytium formation and specific gO genotypes.

UL130 is a 35 kDa glycoprotein, while UL128 and UL131A are smaller, 15 to 18 kDa proteins [116,122,123]. Sequence analyses revealed an extremely high level of conservation of the UL128-UL131A proteins with a mean identity of 98% among clinical isolates [124]. gO and UL128–UL130–UL131A bind to the same site of gH–gL through a disulfide bond with gL-C144. Therefore, they compete for the binding to gL and give rise to alternative types of complexes, the trimeric and the pentameric complexes [123,125,126,127]. It has become widely accepted that the composition of the gH–gL-based complex determines HCMV cell tropism: the gH–gL–gO complex alone is sufficient for HCMV entry and replication in fibroblasts, while the gH–gL–UL128–UL130–UL131A complex extends viral tropism and is required for infection of epithelial, endothelial, and myeloid cells [77,112]. While the pentamer is involved in syncytium formation in epithelial cells and fibroblasts during infection [123,128], the trimer is thought to be involved in cell–cell fusion during fibroblast infection, as syncytia have been observed in these cells also in the absence of pentamer [33,108].

It has been elegantly shown by the group of Ryckman that the relative number of the two alternative gH–gL complexes in the HCMV virion envelope correlates with the infectivity of viral particles and varies greatly among different HCMV strains [129,130]. Therefore, it is tempting to speculate that HCMV strains more prone to syncytium formation might carry substantially different numbers of the two alternative complexes in the virus envelope. Several lines of evidence support an important role of the pentamer in the process of cell–cell fusion. Firstly, the disruption of the disulfide bond existing between UL128 and gL (UL128-C162S/gL-C144S) leads to the impairment of syncytium formation [112]; secondly, the antibody targeting the UL130-131A portion of the pentameric complex results in a complete inhibition of cell–cell fusion [128]; and finally, hypersyncytial variants of TB40/E have been described to contain also a single nucleotide polymorphism in UL128 [131]. Finally, high expression levels of the pentamer have been associated with an increased propensity for cell-associated viral spread and a stricter cell association of the viral progeny [132].

Intriguingly, Calo and colleagues have shown that the HCMV UL116 protein is a non-disulfide bound gH-associated factor alternative to gL and that the gH-UL116 complex is inserted into the viral envelope of mature virus particles [133]. To date, a specific receptor for this glycoprotein complex has not been identified [134], and it is still under debate whether UL116 is part of a new functional gH complex or rather acts as a chaperone that mainly regulates the availability of gH on the virion envelope [135,136]. Though not fully understood, UL116 conservation between herpesviruses and its features reminiscent of adhesion factors suggest an important role in the process of membrane recognition and fusion.

The gM–gN complex is one of the most abundant glycoprotein complexes of the viral envelope. Together with gB, it mediates the attachment of the virions to the cell surface proteoglycans [79]. gM, encoded by ORF UL100, is a 42–45 kDa protein with seven transmembrane domains [137] essential for HCMV replication. gN is encoded by ORF *UL73* and is a highly glycosylated transmembrane protein with an apparent mass of 39–53 kDa [138]. While gM is extremely conserved with 99% mean identity across HCMV strains, the gN coding sequence varies remarkably and is one of the less-conserved proteins with only 81% identity [80]. Although gM–gN are necessary for the initial attachment of viral particles to cellular membranes, which precedes fusion, an involvement of gM–gN in syncytium formation has not been reported thus far [139].

Finally, HCMV encodes four G-protein-coupled receptor homologs (US28, US27, UL33, and UL78) that have been proposed to be important for the recruitment of susceptible cells as well as for the ‘sinking’ of inflammatory chemokines [140,141,142]. US28 is the best characterized of the HCMV GPCR homologs. It is present on the viral envelope and has been shown to enhance the efficiency of cell–cell fusion mediated by viral envelope fusogens [143,144,145]. Even though the molecular mechanism by which US28 facilitates syncytium formation is not known yet, it is intriguing that chemokine receptor signaling activates intracellular Ca^++^ fluxes [146] that in turn have recently been found to play essential roles in cell–cell fusion [23]. However, it is conceivable that high local concentrations of a protein with multiple membrane-spanning domains such as US28 might affect the fluidity or other physical properties of the membrane in a way that is favorable to fusion [144].

## 4. Cellular Determinants and Mechanism of Syncytium Formation

Virus-induced cell–cell fusion necessarily depends on viral proteins as well as properties and proteins characteristic of the host cell. However, our understanding of the cellular determinants of cell–cell fusion is still in its infancy. It is generally assumed that the fusion between virus-infected cells and neighboring cells is triggered by the same proteins involved in the process of virus entry when the viral envelope fuses with the cell membrane. Hence, cells must express the surface molecules that will enable the steps described in Chapter 3. It is reasonable to assume that proteins of the viral fusion machinery expressed on the plasma membrane of one cell can interact with cellular receptors expressed by neighboring cells, activate them, and thereby trigger the fusion of the two plasma membranes. Therefore, the cellular state of fusion competency is, at least in part, determined by the number and distribution of the receptors and molecules engaged by the viral fusion machinery. For example, the epidermal growth factor receptor (EGFR) plays an important role in (hyper-)fusogenicity of HSV, and its overexpression increases the formation of multinucleated giant cells [147]. Although subsequent work has indicated that EGFR is more likely to function as a co-receptor [148], EGFR has initially been proposed as an HCMV entry receptor [149]. HCMV-induced signaling through the EGFR has been recognized as a key control point for virus–host cell interaction and regulation of both viral and cellular activities [150]. In addition to EGFR, other receptors such as the platelet-derived growth factor receptor alpha (PDGFRα), Neuropilin-2, olfactory receptor family 14 subfamily I member 1 (OR14I1), and specific integrins and adhesion molecules have been shown to be crucial for HCMV entry [151,152,153]. Some of these proteins also play a role in the physiological and virus-independent formation of multinucleated giant cells [154]. However, their involvement in virus-induced cell fusion has not been elucidated. These molecules are expressed on the surface of many different cell types and activate several intracellular pathways that govern a wide variety of processes such as cell growth and survival, migration, and adhesion. Indeed, fusion competence requires that cells move toward one another, change their cytoskeletal architecture and shape, and finally loosen the intervening membrane to undergo cytoplasmic mixing without the activation of programmed cell death [9,155]. Overall, the cellular players and mechanisms leading to syncytium formation are probably numerous and diverse, depending not only on the specific cell type but also on the activation/differentiation state of the ‘fusion-competent’ cell [156,157]. For instance, while human foreskin fibroblasts (HFF) have been reported to be barely able to fuse, human fetal lung fibroblasts (MRC-5 cells) exhibit a much higher propensity to fuse upon HCMV infection [108,158]. These observations suggest that unrecognized cellular factors must play major roles in the formation of virus-induced syncytia.

## 5. Role of Syncytia in Viral Pathogenesis

Even though multinucleated syncytia were first described in HSV and VZV lesions in patients in the early fifties, their significance for replication and spread of the virus in vivo has remained largely unclear. The enormous variation in the degree of cell fusion produced by different clinical and laboratory strains in tissue cultures [159] has contributed to the debate of whether these multinucleated cells are a natural phenomenon or instead an in vitro artifact. Often HSV-induced syncytia found both in lesions and in tissue culture contain no more than 10 nuclei, whereas viral variants causing much more extensive fusion in tissue culture and the appearance of syncytia with hundreds of nuclei are readily isolated from high titer laboratory stocks or upon in vitro passaging. Several studies have shown that the ability to form syncytia is a pathogenicity factor in alphaherpesvirus infections [42]. For example, the fusion between nerve cells and HSV, VZV, or pseudorabies virus-infected epithelial cells has been reported to cause increased electrical activity of the neuronal cells and to be correlated with the peripheral neuropathies, itching sensations, and persistent pain associated to these two infections [160]. Moreover, following footpad inoculation of mice, some hyperfusogenic HSV strains have been shown to induce a striking alteration in the infection pathogenesis in vivo and to cause dramatic acute inflammatory responses and even paralysis of the inoculated limb [98].

In contrast to HSV, research on syncytium formation induced by HCMV is still in an early stage. HCMV has for a long time been considered a virus that is not or only rarely syncytium-forming. However, this assumption is probably biased, as the most commonly used cell type for HCMV propagation, human dermal fibroblast, is rather resistant to cell–cell fusion. Moreover, the common method of virus isolation from patient samples, based on the inoculation of fibroblast monolayers with biological fluids such as urine, saliva, bronchoalveolar lavage fluid, or blood, has complicated rather than supported a better understanding of HCMV-induced syncytium formation and its frequency in clinical HCMV strains. More recent studies, primarily with ARPE-19 epithelial cells, have revealed that some HCMV strains or isolates can be highly fusogenic and induce the formation of very large syncytia [123,128,131]. Hence, syncytium formation by HCMV should be studied by infecting fusion-competent cells such as ARPE-19 and MRC-5 rather than HFF.

Recent studies reported syncytium-forming phenotypes and genetic association among clinical HCMV isolates obtained from congenitally or postpartum infected newborns [54,161]. These observations raise the question of whether syncytium-forming HCMV strains might be associated with increased transmission, virulence, or pathogenicity. HCMV primary infection during pregnancy results in an approximately 30–40% chance of transmission to the fetus. Approximately 15% of congenitally infected infants suffer from acute disease or late sequelae such as sensorineural hearing loss or mental retardation [29]. The causative factors, as well as the underlying molecular mechanisms, remain largely unknown [162]. In the light of recent findings, it seems likely that, besides immunological factors (i.e., the presence of neutralizing antibodies and reactive T cells), specific properties of the virus also play a decisive role. A highly reactive fusion machinery might allow HCMV to infect certain cells and tissues more efficiently, to cross tissue barriers such as the placental barrier and the blood–brain barrier, and to spread more easily in the presence of neutralizing antibodies.

## 6. Conclusions and Future Perspectives

HCMV and other betaherpesviruses are, in principle, capable of inducing cell–cell fusion and the formation of syncytia. Although our understanding of the viral and cellular factors regulating cell–cell fusion is very incomplete, it is clear that, among the viral factors, the envelope glycoproteins play a crucial role. Glycoprotein B, the viral fusogen, is essential for the fusion of the viral envelope with host cell membranes as well as for the fusion of infected and neighboring cells. Indeed, gB variants associated with increased fusogenicity and syncytium formation have already been described. Besides gB, the glycoproteins involved in receptor binding and activation of gB are likely to play an equally important role: the trimeric (gH–gL–gO) and the pentameric (gH–gL–UL128–UL130–UL131) glycoprotein complexes. However, specific variants associated with increased formation of syncytia have yet to be identified. Such variants could promote syncytium formation either by facilitating gB activation or by increasing the level of expression or cell surface presentation of the respective gH–gL complex. To what extent other viral proteins influence cell–cell fusion remains to be investigated.

To date, very little is known about the cellular factors involved in cell fusion. Some cell types are more susceptible to HCMV-induced syncytia formation (e.g., ARPE-19 cells and MRC-5 cells), others appear to be more resistant (e.g., HFF and HUVEC), and many others have not been tested (e.g., myeloid cells). Future studies will show whether the susceptibility to virus-induced cell–cell fusion depends on the expression levels of virus entry receptors on the cell surface, on cell morphology and changes induced by viral infection, or on other factors yet to be defined.

While it is clear that HCMV strains and variants with different levels of fusogenicity exist, it is much more difficult to determine whether the ability to induce cell–cell fusion is a pathogenicity factor in the human host. Maybe HCMV variants with a highly active fusion machinery are more capable of infecting certain cell types and crossing tissue barriers in vivo. Recent reports of syncytium-forming HCMV variants isolated from congenitally infected newborns support the hypothesis that syncytial variants might indeed play a role in transmission and pathogenesis. However, many more studies are needed for a comprehensive understanding of the molecular determinants of cell–cell fusion in cell culture and the importance of syncytial variants for transmission and disease.

## Data Availability

Not applicable.
